# Localization of Radio Sources Using High Altitude Platform Station (HAPS)

**DOI:** 10.3390/s25061935

**Published:** 2025-03-20

**Authors:** Yuta Furuse, Gia Khanh Tran

**Affiliations:** Department of Electronic Engineering, Institute of Science Tokyo, 2-12-1 Ookayama, Meguro-ku, Tokyo 152-8550, Japan

**Keywords:** HAPS, MUSIC, DOA

## Abstract

In Japan, the DEURAS system has been deployed to detect and locate illegal radio sources that either exceed permissible transmission power limits or operate on unauthorized frequencies. This system utilizes receiving antennas installed on high-rise buildings and radio towers to capture radio signals and estimate the location of the transmission source. However, in densely built urban environments, the accuracy of location estimation is often compromised due to signal reflections and diffractions. Additionally, in large-scale disasters such as earthquakes, terrestrial infrastructure may be severely damaged, making it essential to develop a localization system that operates independently of ground-based stations. To overcome these limitations, this study proposes a localization system based on a high-altitude-platform station (HAPS), which operates at an altitude of approximately 20 km. The feasibility and effectiveness of the proposed system are evaluated through numerical simulations, considering various environmental conditions. The results demonstrate that HAPS-based localization significantly improves positioning accuracy, offering a robust and high-precision alternative for radio source detection, particularly in scenarios where traditional ground-based systems are unreliable or unavailable.

## 1. Introduction

### 1.1. Background and Objectives

Japan has over 300 million radio stations, and the number continues to increase each year. As of 2022, land mobile stations, including mobile phone terminals, accounted for 98.9% of all radio stations, indicating the widespread use of mobile communication devices [1]. However, unauthorized radio stations that exceed power limits or use illegal frequencies have been confirmed to exist [2], necessitating appropriate countermeasures.

Currently, the DEURAS radio monitoring system is in operation to monitor and crack down on illegal radio stations. This system uses fixed sensor stations installed across various locations and vehicle-mounted sensor stations, which are remotely controlled by central stations located in regional communication bureaus. These sensors monitor radio waves, measure the direction of radio sources, and identify the locations of illegal radio stations [3]. However, in dense urban environments with numerous high-rise buildings, direct line of sight between sensor stations and illegal radio stations may be obstructed, and the presence of multiple reflected waves may hinder accurate location estimation. Additionally, the monitoring range of a single sensor station is limited, and as of 2018, approximately 350 sensor stations were deployed [4].

In recent years, high-precision localization methods using UAVs (unmanned aerial vehicles), such as drones, in non-line-of-sight environments have been proposed but have not yet been implemented [5]. Furthermore, accurate localization of radio transmission sources over a wide area could contribute to rapid rescue operations in the event of a disaster when ground base stations become inoperative.

This paper aims to address these challenges by utilizing a HAPS (high-altitude-platform station) [6,7,8,9]. A HAPS refers to a system that operates unmanned aerial vehicles, such as aircraft flying at approximately 20 km in the stratosphere, as communication base stations to provide communication services over wide areas. By leveraging a HAPSs for radio source localization, it is expected that the impact of reflected waves can be reduced due to its high-altitude operation, allowing for wider radio monitoring and localization compared to conventional monitoring systems. This paper explores the use of HAPSs for monitoring illegal radio transmissions and estimating the location of mobile terminals during disasters. The effectiveness of this approach is demonstrated primarily through numerical simulations performed using MATLAB R2024a [10]. Additionally, instead of aiming for pinpoint localization of illegal radio transmissions, this study focuses on narrowing down the general transmission area. This approach assumes that after the localization by a HAPS, an illegal radio detection vehicle will be used for more precise identification.

### 1.2. Related Research

Various studies have been conducted on radio source localization. In a study in [5], a method using UAVs, such as drones, estimates the location of radio sources by leveraging pre-trained data, including signal strength. This approach achieves high localization accuracy even in environments with poor line of sight to the transmission source. Additionally, in a study in [11], a location fingerprinting method was used for localization in a simulated outdoor environment, demonstrating that optimizing the UAV’s trajectory improves estimation accuracy. However, these studies focus on localization within outdoor environments or university campuses, which does not address the challenges faced by the current DEURAS radio monitoring system in large-scale search areas. Moreover, large-scale exploration requires an extensive amount of training data.

Interestingly, ref. [12] explored the integration of artificial intelligence (AI) into radio localization techniques for outdoor environments. The authors introduced LocUNet, a convolutional neural network designed for real-time outdoor localization using received signal strength (RSS) from base stations. Unfortunately, machine learning-based approaches require high computation and storage complexity for both the training and estimation phases, which is not suitable for the large-scale scenarios considered in this paper.

In the studies in [13,14], experiments were conducted on the localization of ground-based radio sources using high-altitude unmanned aircraft. Both studies utilized direction-of-arrival (DOA) estimation and demonstrated high accuracy. However, these experiments were conducted in environments with clear line of sight and no obstacles. Additionally, the maximum altitude tested was only about 3 km, leaving the effectiveness of localization at higher altitudes unverified.

In contrast, our previous research [15] explored ground-based radio source localization using HAPSs at an altitude of 20 km. However, this study was limited to simulations with a single transmission source and did not consider multiple sources. Furthermore, although multiple HAPS units were used for localization, it is unlikely that multiple HAPSs would be available simultaneously for a single transmission source when considering their coverage as communication base stations. Therefore, in this study, we focus on localization using a single HAPS.

As outlined above, previous research has not thoroughly addressed localization in wide-area, obstacle-dense environments. To address this gap, this paper demonstrates through simulations that using a HAPS enables high-accuracy localization, even in urban environments with dense buildings and large coverage areas.

## 2. Materials and Methods

### 2.1. Antenna Configuration

The antenna specifications used in the localization simulation are shown in Table 1.

**Figure 1:**
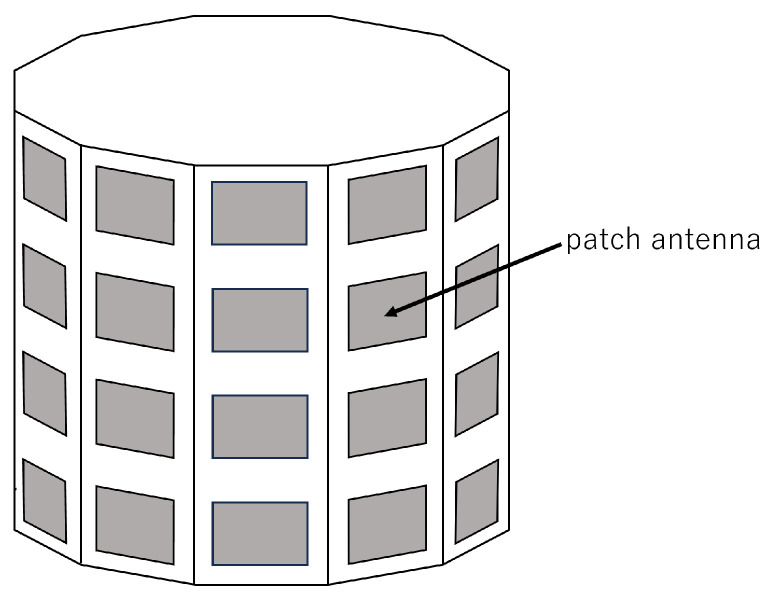
Schematic diagram of the cylindrical antenna.

The reception antenna used in the simulation was modeled as a cylindrical antenna, as employed when utilizing a HAPS as a base station [16]. The basic structure of the cylindrical antenna is shown in Figure 1. In reality, the antenna consists of 31 elements arranged in a circular configuration and 6 elements stacked vertically, but it is simplified for representation.

**Figure 2:**
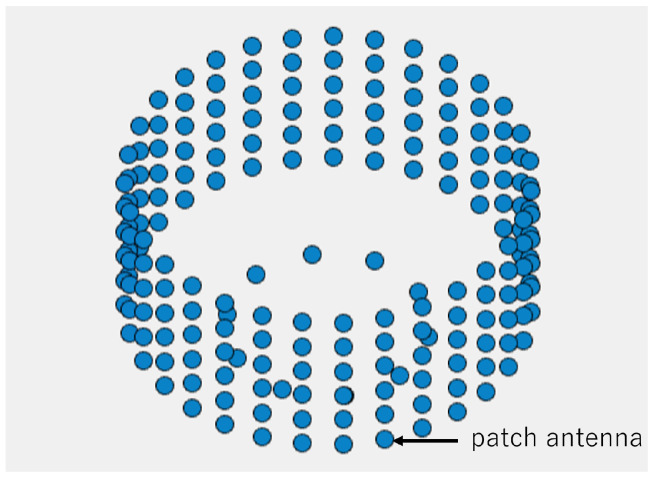
Cylindrical antenna used in the simulation.

**Figure 3:**
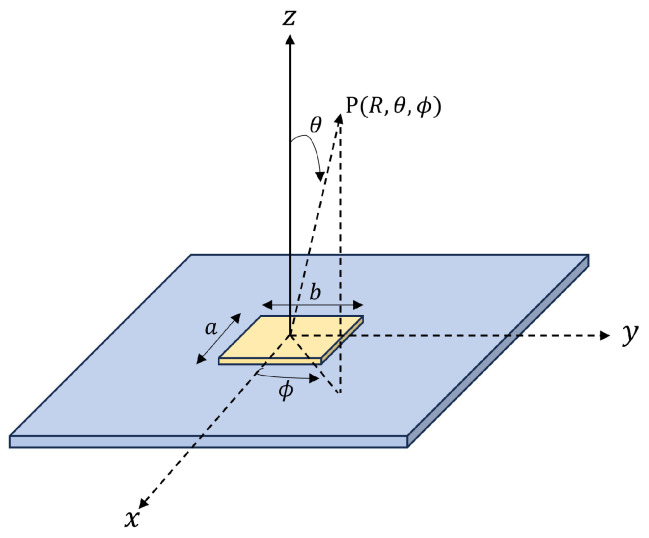
Patch antenna.

For the simulation, an antenna with elements arranged as shown in Figure 2 was used. The specifications of the cylindrical antenna are listed in Table 2. Each element is a patch antenna with directivity, as illustrated in Figure 3. In Table 2, λ represents the signal wavelength.

The reception pattern of the patch antenna is given by the following theoretical formula [17]. $\epsilon_{r}$ represents the permittivity.

(1) $$E_{\theta }=-jKf\left(\theta ,\phi \right)\cos\phi \frac{\epsilon_{r}-\sin^{2}\theta }{\epsilon_{r}-{\left(\sin\theta \cos\phi \right)}^{2}}$$

(2) $$E_{\phi }=-jKf\left(\theta ,\phi \right)\cos\theta \sin\phi \frac{\epsilon_{r}}{\epsilon_{r}-{\left(\sin\theta \cos\phi \right)}^{2}}$$

f(θ,ϕ) and the constant *K* are given by the following equations. Additionally, *a* and *b* represent the length and width of the antenna element, respectively, as shown in Figure 3. $V_{0}$ is a value determined by the feeding voltage, *R* is the distance from the antenna to the observation point, and $k_{0}$ is the wavenumber of a plane wave in free space.

(3) $$f\left(\theta ,\phi \right)=\left(\frac{\sin u}{u}\right)\cos v$$

(4) $$u=\left(\frac{k_{0}b}{2}\right)\sin\theta \sin\phi$$

(5) $$v=\left(\frac{k_{0}a}{2}\right)\sin\theta \cos\phi$$

(6) $$K=\left(\frac{V_{0}k_{0}b}{\pi }\right)\left(\frac{\exp(-jk_{0}R)}{R}\right)$$

In this simulation, the relative permittivity was set to $\epsilon_{r}=2.6$, with the element size in the *x*-direction as a=0.5λ and in the *y*-direction as b=0.6λ. Using these parameters, the directivity gain can be expressed as follows.

(7) $$G_{d}\left(\theta ,\phi \right)=\left(\frac{{4\pi |E\left(\theta ,\phi \right)|}^{2}}{\int_{0}^{2\pi }d\phi \int_{0}^{\pi /2}{\left|E\left(\theta ,\phi \right)\right|}^{2}\sin\theta \;d\theta }\right)$$

**Figure 4:**
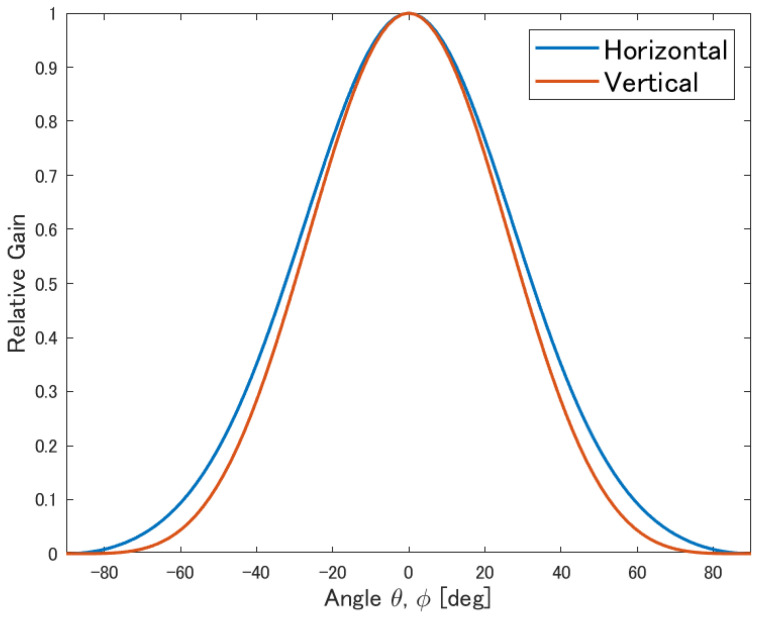
Antenna pattern.

The total electric field magnitude is given by ${\left|E\left(\theta ,\phi \right)\right|}^{2}=|E_{\theta }{\left(\theta ,\phi \right)|}^{2}+{\left|E_{\phi }\left(\theta ,\phi \right)\right|}^{2}$. The normalized reception pattern obtained from these equations is shown in Figure 4. The maximum gain was adjusted to 8 dBi to ensure consistency in the simulation results.

**Figure 5:**
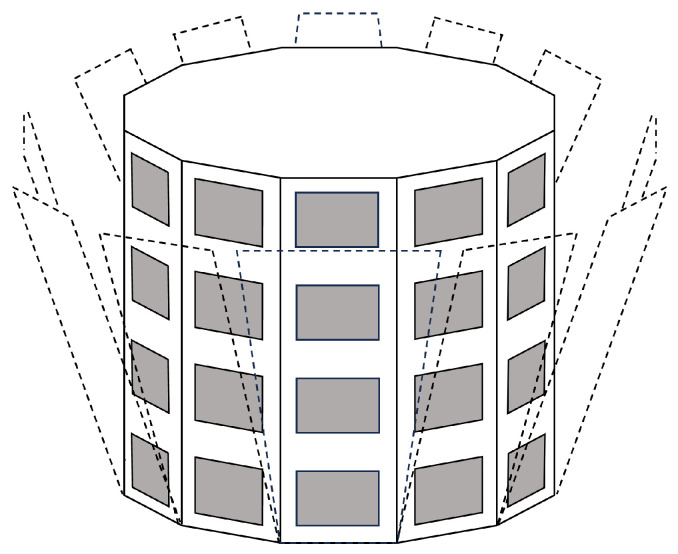
Cylindrical antenna with tilted element plate.

Additionally, to ensure that the antenna can direct its beam toward transmission sources located below the HAPS, a modified antenna configuration was also used in the simulation. As shown in Figure 5, this was achieved by tilting the element panel with attached patch antennas, thereby directing the beam downward.

### 2.2. Radio Propagation Model

In this study, ray-tracing simulation was used to estimate radio-wave propagation, and therefore statistical models were not applied. When considering a radio-wave propagation model with a HAPS as the receiving station, various propagation losses must be taken into account. These include free-space path loss, gaseous absorption and rain attenuation, terrain-induced losses, vegetation losses, building penetration losses, clutter losses due to building obstructions, human body obstruction losses, and diffraction losses due to spherical Earth curvature. The free-space path loss was calculated using the Friis formula, as shown below.

(8) $$PL_{\mathrm{fsp}}=10\log_{10}{\left(\frac{4\pi d}{\lambda }\right)}^{2}\;\left[\mathrm{dB}\right]$$

*d* and λ represent the distance between the transmitting and receiving antennas and the wavelength, respectively. The attenuation due to gaseous absorption and rainfall was not considered in this study, as the ITU-R recommendations [18,19] indicate that their impact is negligible at 2 GHz. Since the simulation assumed an urban environment with dense buildings, terrain-induced diffraction and vegetation losses were also not considered. Building penetration loss was not considered because the radio sources targeted for localization were assumed to be located outdoors. The losses caused by building obstructions were attributed to reflection and diffraction. In this study, simulations were primarily performed considering only reflected waves. Additionally, another simulation was performed taking into account edge diffraction, where radio waves refract upon incidence on building edges (corners and boundaries). Human body obstruction loss was not considered to simplify the analysis. The effects of spherical Earth diffraction were also not considered, as the receiving point was not located near the Earth’s surface.

Based on the above considerations, the propagation losses accounted for in this simulation include free-space path loss, building reflection, and ground reflection for the purpose of localization. The attenuation due to ground and building reflections, denoted as $L_{\mathrm{ref}}$, was calculated using the Fresnel equations in the ray-tracing simulation. The relative permittivity and conductivity of the surface materials used in this analysis were referenced from ITU-R studies [18]. Both the ground and buildings were assumed to be made of concrete, with a relative permittivity of 5.31 and a conductivity of 0.0548 S/m. Considering these factors, the total propagation loss PL was computed as follows.

(9) $$PL=PL_{\mathrm{fsp}}+PL_{\mathrm{ref}}\;\left[\mathrm{dB}\right]$$

### 2.3. Signal Processing System

The received signal at the antenna is generated based on the propagation characteristics. For simplicity, we considered a scenario where a single transmission source exists, and *L* incoming waves arrive at the receiving antenna. Using the propagation distance and the number of reflections obtained from the ray-tracing simulation, the propagation loss PL[dB] for each incoming wave (l=1,2,…,L) was calculated using Equation (9). The input power of each incoming wave was then determined using the following equation.

(10) $$P_{l}=P_{\mathrm{source}}\times 10^{-\left(\frac{PL_{l}}{10}\right)}\;\left[W\right]\;\left(l=1,2,\ldots ,L\right)$$

$P_{\mathrm{source}}$ represents the transmission power of the source. Furthermore, using the phase shift Δϕ(l=1,2,…,L) obtained from the ray-tracing simulation, the complex signal of the l-th wave was calculated using the following equation.

(11) $$F_{l}\left(t\right)=\sqrt{P_{l}}\exp\left(2\pi ft+\Delta \phi_{l}\right)\;\left(l=1,2,\ldots ,L\right)$$

The steering matrix A for each element of the cylindrical antenna was calculated using the steervec function in MATLAB R2023a’s Phased Array System Toolbox. The thermal noise power $P_{N}$ generated at the receiving antenna was calculated using the following equation.

(12) $$P_{N}=10\log_{10}\left(kTB\right)+NF\;\left[\mathrm{dBW}\right]$$

k,T,B, and NF represent Boltzmann’s constant, the absolute temperature of the noise source, the bandwidth, and the noise figure, respectively. In this study, the absolute temperature at an altitude of 20 km was set to 216.5K, and the bandwidth was assumed to be 10MHz for the simulation. The noise figure was set to 3dB, based on previous research on HAPS systems [20].

Using the signal of each incoming wave $F_{l}$, the steering matrix A, and the thermal noise power $P_{N}$, the input vector X(t) at each antenna was calculated using the following equation.

(13) X(t)=AF(t)+N(t)

(14) $$F\left(t\right)={\left[F_{1}\left(t\right),F_{2}\left(t\right),\ldots ,F_{L}\left(t\right)\right]}^{T}$$

N(t) represents the thermal noise vector, where each component is an independent complex Gaussian process with a mean of 0 and a variance of $P_{N}$. By substituting random values for *t* in Equation (13), the generated signals were used as the complex input signals for each array element. Here, the number of substitutions corresponds to the number of samples. For the obtained input vector X(t), the MUSIC method [21] was applied to estimate the direction of arrival (DOA) of the incoming signals. Additionally, the Akaike information criterion (AIC) [22] method was used to estimate the number of incoming waves.

### 2.4. Location Estimation Method

**Figure 6:**
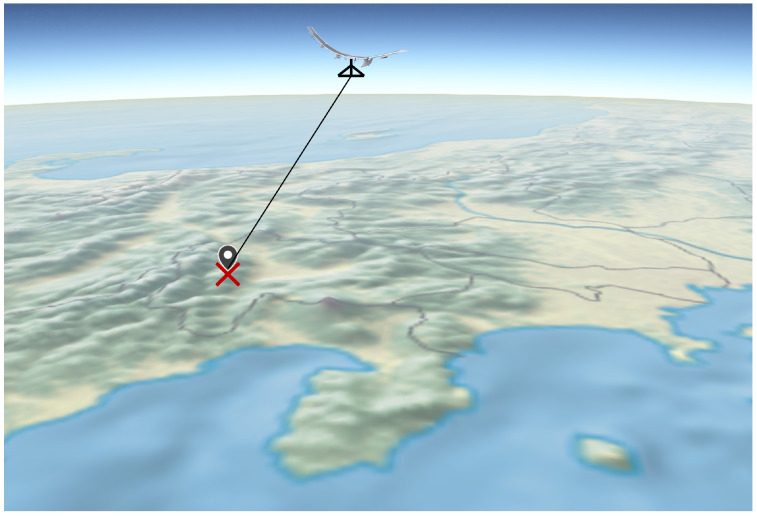
Estimated coordinate determination method.

In this simulation, a single HAPS was used to estimate the direction of arrival (DOA) of the radio source. The HAPS’s own position was assumed to be accurately obtained via GPS or similar means. Using the HAPS’s position along with the estimated elevation and azimuth angles, a straight line was generated in a three-dimensional space. The intersection of this line with a model where the Earth is approximated as a sphere was considered the estimated position of the radio source. A schematic diagram illustrating this concept is shown in Figure 6.

The height of the spherical surface, representing the estimated region where the radio source exists, can be derived based on the estimated elevation and azimuth angles. Ideally, this height could be obtained from average terrain elevation data. However, in this simulation, for simplicity, a fixed value was used for calculation. When performing localization using HAPSs, compared to ground-based antenna DOA estimation, the elevation angle is often close to vertical relative to the ground. This characteristic helps minimize horizontal positioning errors even if there is some uncertainty in the source height. Therefore, this approach was adopted in the simulation. Additionally, if neither the direct wave nor the reflected wave (and in some cases, the diffracted wave) reaches the HAPS, or if the received signal power is too low to determine a reliable location, the estimation is considered unsuccessful.

Furthermore, as shown in Section 2.3 and Section 2.4, our method merely uses fundamental linear algebra, that requires no specific additional computation resource. Such computation can be performed onboard at the HAPS and the estimation results then can be sent to the ground-based control center via the communication link (feeder link) between the HAPS and the ground station.

## 3. Results

This section describes the localization simulations performed in this study. The simulations were performed for cases where a single stationary radio source and multiple stationary radio sources exist. Additionally, the number of sources was assumed to be unknown, and localization was carried out using the MUSIC (Multiple Signal Classification) method.

### 3.1. Single Radio Source Localization

**Figure 7:**
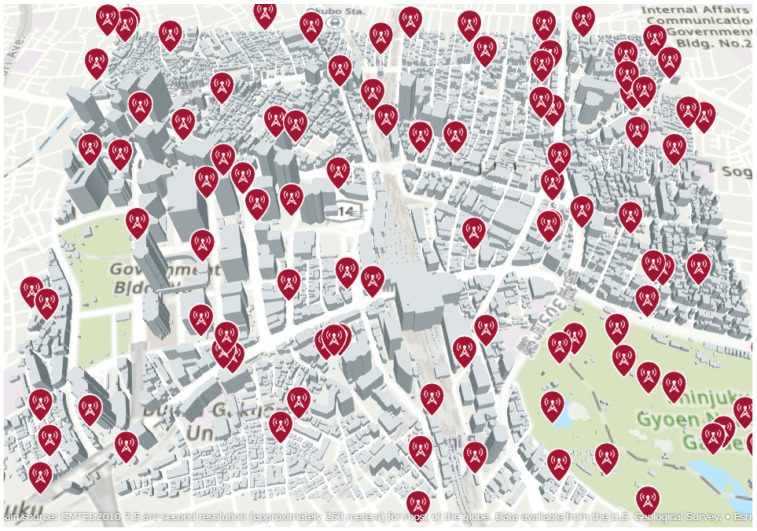
Radio sources placed randomly in the environment.

**Figure 8:**
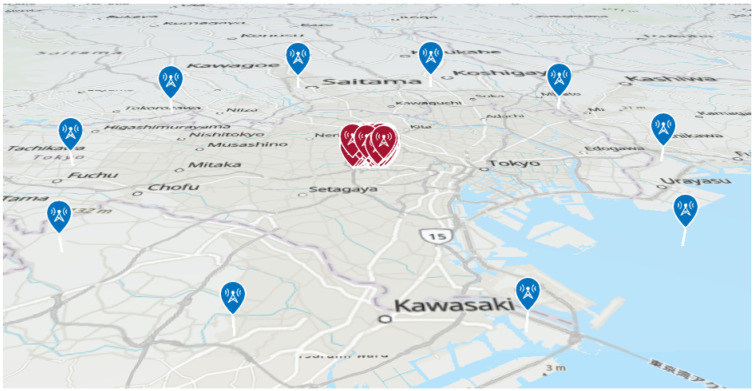
HAPS placement at a fixed distance from the urban area.

As shown in Figure 7, the urban environment of Shinjuku was used as the simulation model for localization. A single radio source located within this environment was considered for estimation. The position of the transmission source was varied across 1000 different patterns, while the HAPS was placed at 10 different locations, each maintaining a constant horizontal distance from the center of the Shinjuku 3D environment, as shown in Figure 8. Localization was performed for each HAPS placement. The transmission power of the source was set to 10 W, and the distance between the center of Shinjuku and the HAPS was varied from 5 km to 100 km to evaluate the accuracy of the localization.

**Figure 9:**
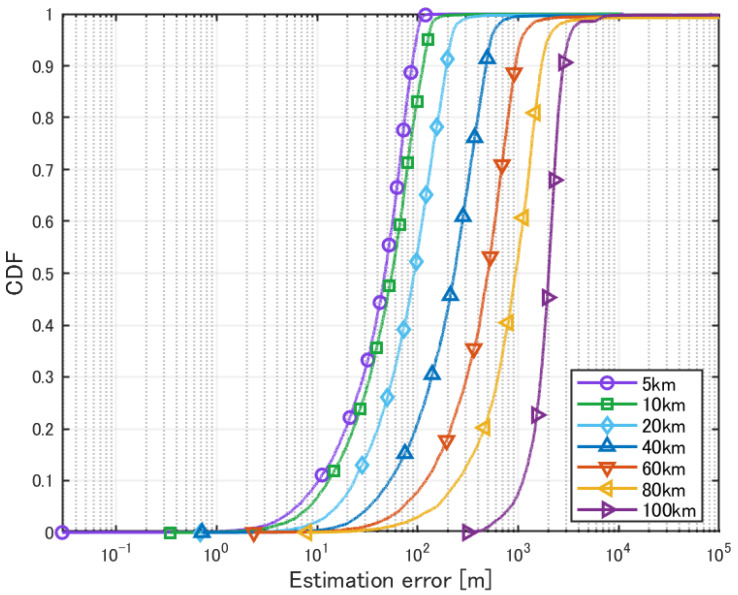
Cumulative distribution of localization error for a single source.

The obtained evaluation results are presented in Table 3 and Figure 9.

The detection percentage represents the ratio of successful localizations out of 10,000 trials. The mean error and CDF 90% were calculated only for cases where a valid estimated position was obtained.

**Figure 10:**
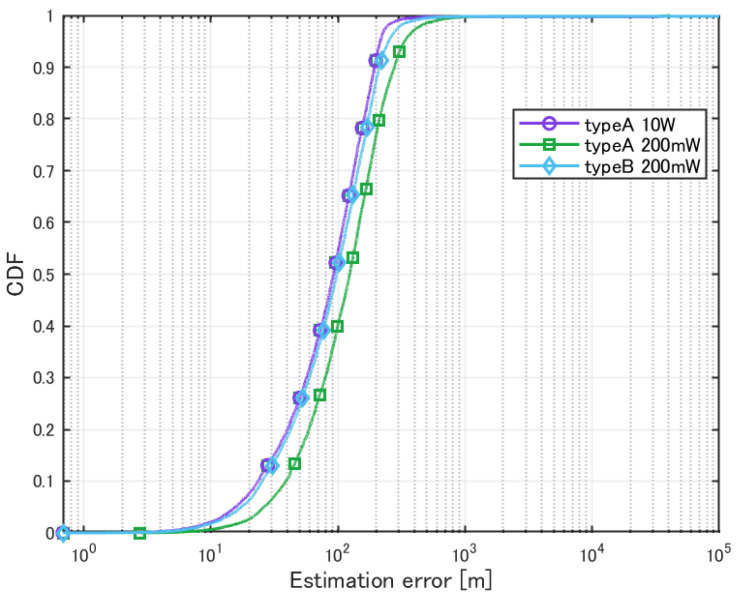
Cumulative distribution of localization error for a low-power single source.

Next, localization was performed for a low-power transmission source under the same conditions. In this case, the transmission power of the source was set to 200 mW. In addition to the standard cylindrical antenna, another simulation was performed using a modified antenna, where the antenna elements were tilted downward by 45° from the horizontal plane (0°), as shown in Figure 5. These localization simulations were performed only for the case where the HAPS was positioned 20 km away from the center of the Shinjuku 3D environment. The obtained results are summarized in Table 4 and Figure 10. The standard cylindrical antenna (without element tilt) is defined as type A, while the tilted antenna (elements tilted by 45°) is defined as type B. For comparison, the results from the previous case with a 10 W transmission source are also included. To ensure meaningful evaluation, cases where the estimation error exceeded 10 km, indicating a failure in identifying the transmission source, were excluded from the analysis.

**Figure 11:**
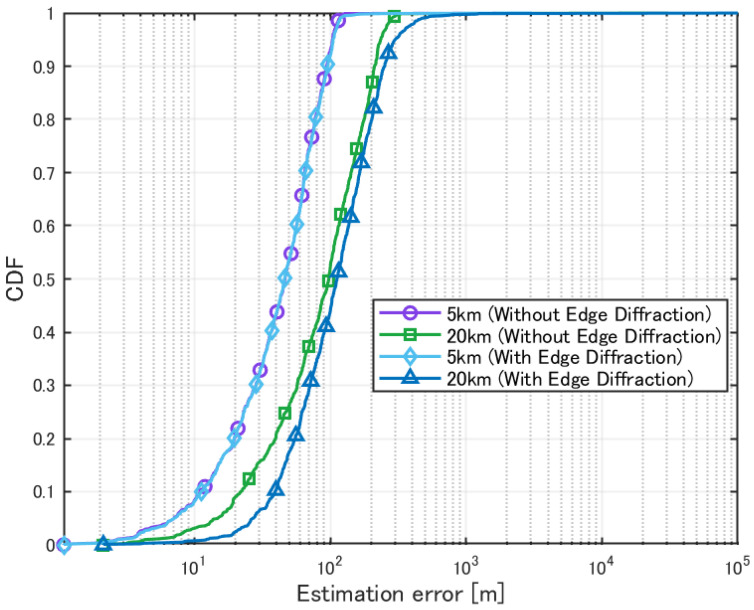
Cumulative distribution of localization error for a single source with edge diffraction.

Next, a localization simulation was performed while considering edge diffraction effects. The transmission power of the source was set to 10 W, and the number of source location patterns was set to 100. The horizontal distance from the 3D environment to the HAPS was set to 5 km and 20 km, and localization was performed for these two cases. The evaluation results are presented in Table 5 and Figure 11. As seen from this figure, we found out that the angle of arrival changed insignificantly when edge diffraction was taken into account, such that the relative degradation of localization accuracy (compared to the absolute value of the error) was not significant. For this reason, analysis of edge diffraction was just partially conducted in this paper.

### 3.2. Multiple Radio Source Localization

**Figure 12:**
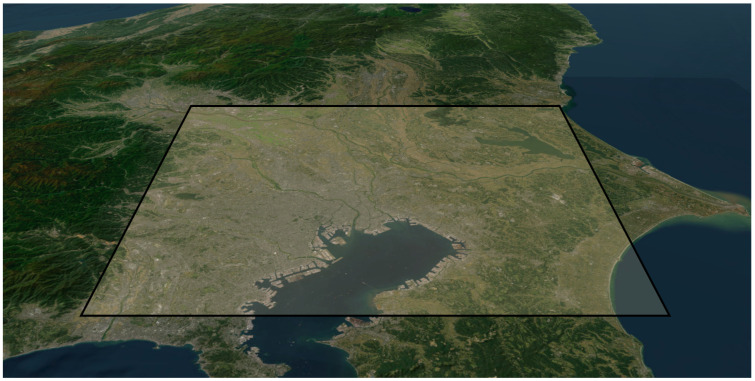
Transmission source generation area.

As shown in Figure 12, multiple transmission sources were simulated within a 100 km × 100 km area. However, since the area is extensive, building obstructions were not considered, and only terrain effects were included in the simulation. For propagation modeling, only the direct wave and ground reflection were taken into account.

**Figure 13:**
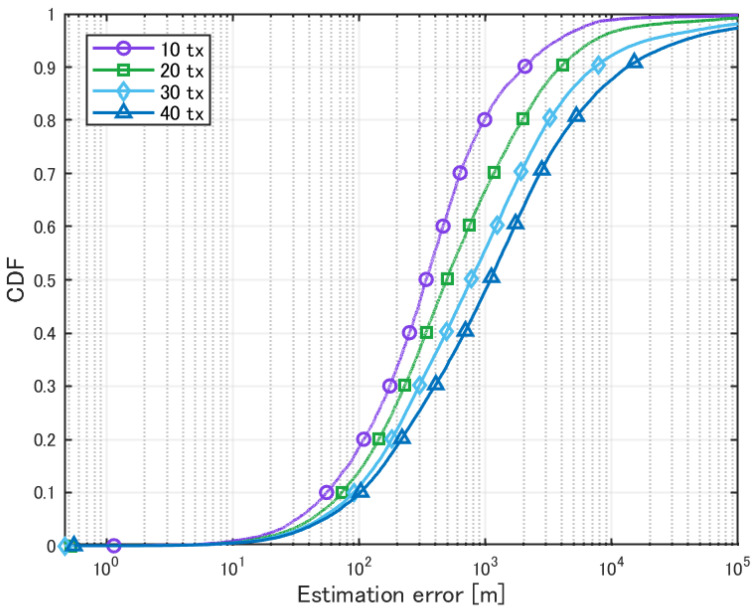
Cumulative distribution of localization error for multiple sources.

The antenna used was the same as the one shown in Figure 5, tilted 45° downward. The HAPS was positioned at the center of the area, and localization was performed. The evaluation results for different numbers of transmission sources are presented in Table 6 and Figure 13. For each case, 1000 simulation patterns were analyzed.

**Figure 14:**
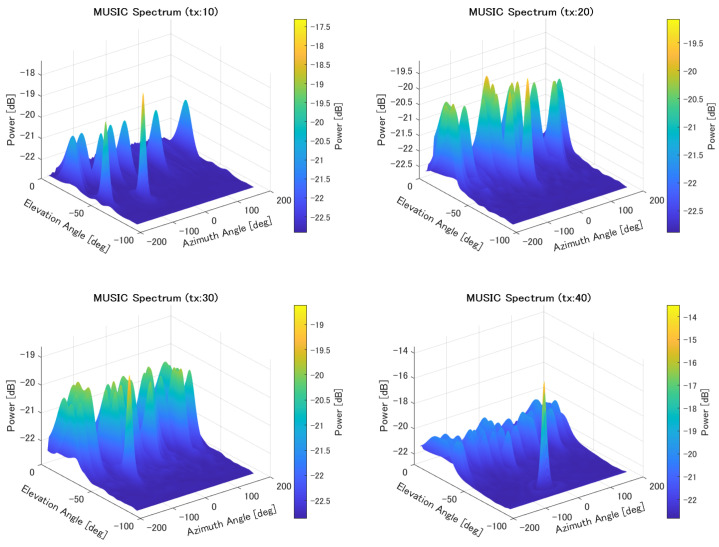
MUSIC spectrum for multiple sources.

The MUSIC spectrum for localization corresponding to each number of transmission sources is presented in Figure 14. These spectra illustrate the estimated DOA peaks, indicating the detected directions of incoming signals for different source configurations.

**Figure 15:**
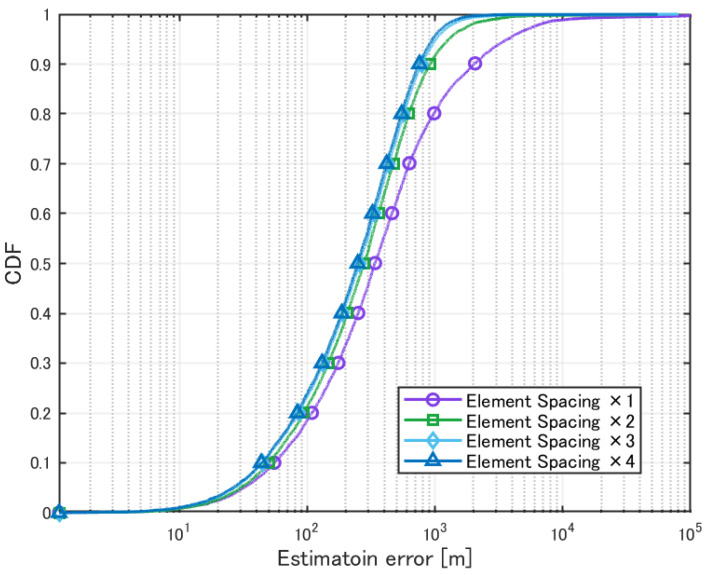
Cumulative distribution of localization error for multiple sources with varying element spacing.

Next, the evaluation results for the case where 10 transmission sources were present are presented. The element spacing was varied from 1 to 4 times the reference spacing, and the impact on localization accuracy was analyzed. The obtained results are summarized in Table 7 and Figure 15.

**Figure 16:**
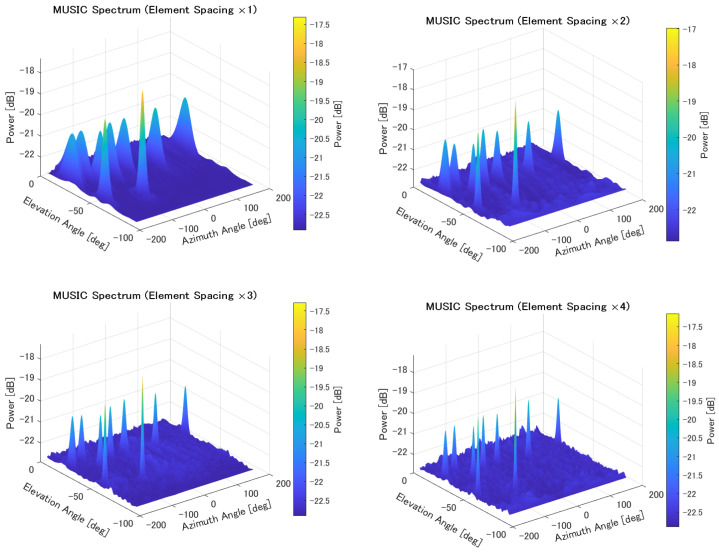
MUSIC spectrum for multiple sources with varying element spacing.

The MUSIC spectrum obtained for each element spacing is shown in Figure 16.

**Figure 17:**
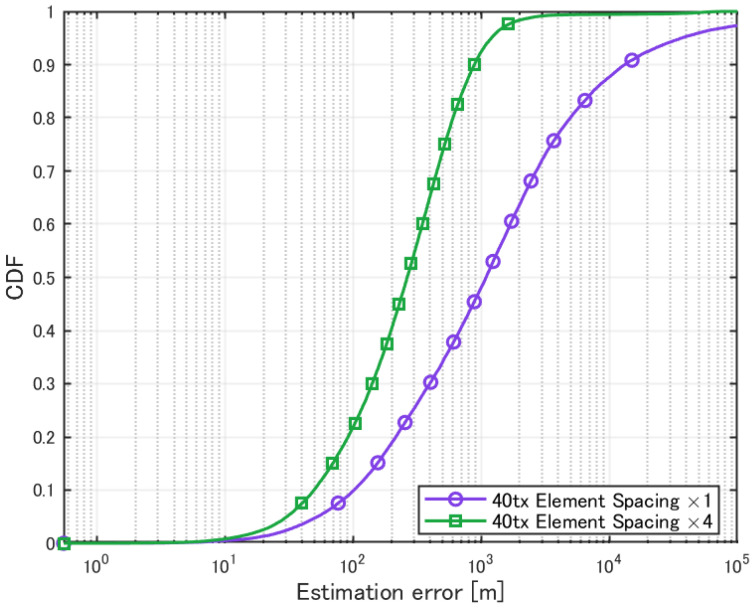
Cumulative distribution of localization error with varying element spacing (40 transmission sources).

Additionally, simulations were performed in a scenario with 40 transmission sources, where the element spacing was varied. The evaluation results are presented in Table 8 and Figure 17.

**Figure 18:**
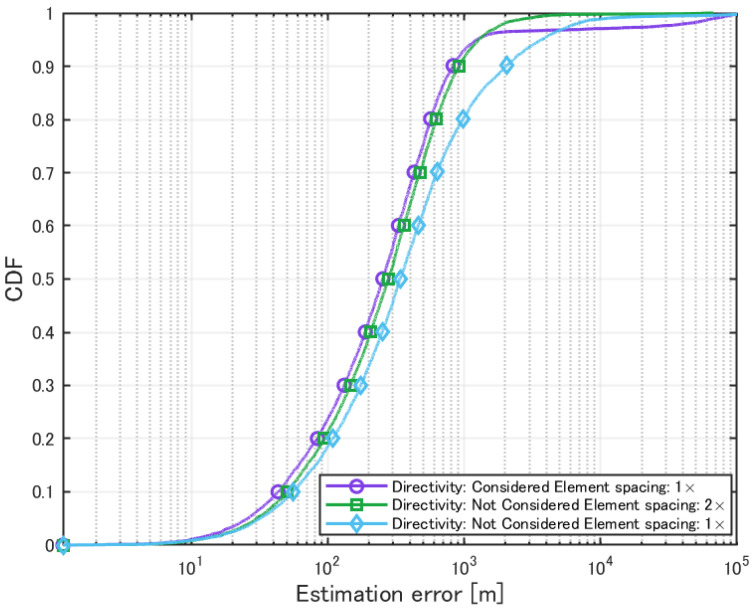
Cumulative distribution of localization error with the MUSIC method considering element directivity.

Next, localization simulation was performed by incorporating the directional elements of each antenna element into the steering vector of the MUSIC spectrum. The number of signal sources was set to 10, and the antenna element spacing was set to the standard interval. The evaluation results are shown in Table 9 and Figure 18. For comparison, we also present the results obtained using a standard antenna without considering element directivity, as well as the results when the element spacing was doubled without considering element directivity.

**Figure 19:**
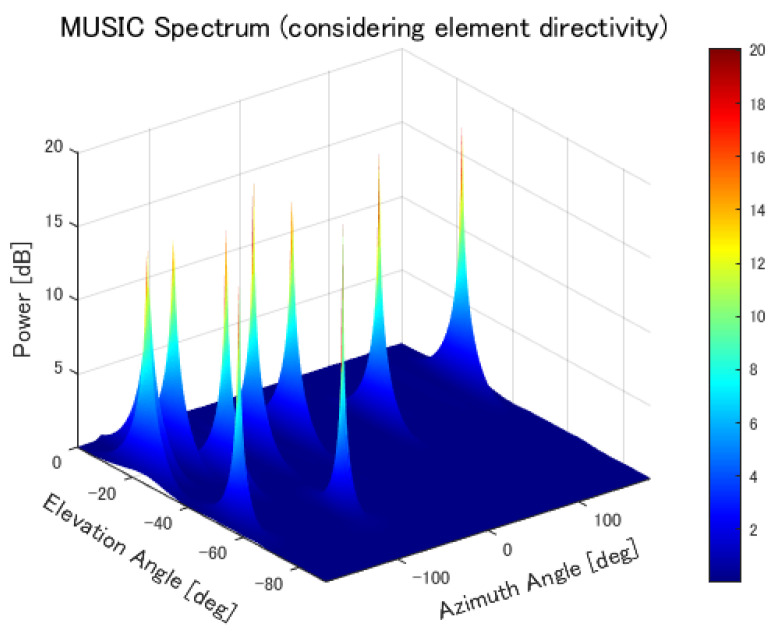
MUSIC spectrum with element directivity considered.

The MUSIC spectrum considering element directivity is shown in Figure 19.

**Figure 20:**
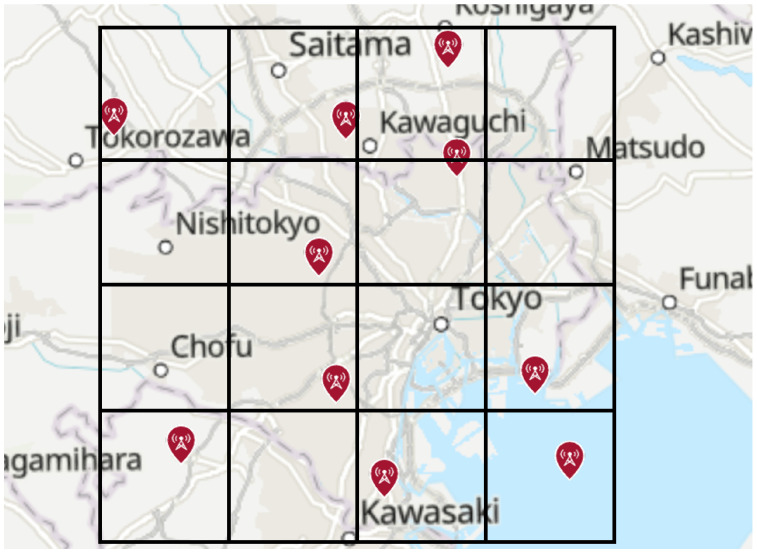
Sources placement assuming cells (cell side length: 10 km).

Next, assuming localization for a mobile terminal, the transmission power of the signal source was set to 200 mW and localization performed based on the cell deployment of base stations, as shown in Figure 20. As in the previous scenario, the influence of buildings was not considered.

**Figure 21:**
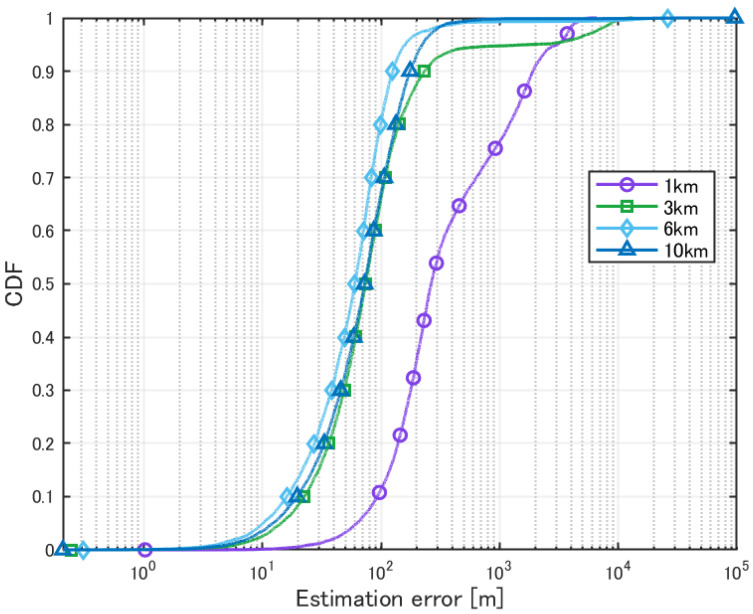
Cumulative distribution of localization error in cell-based deployment.

The side length of each cell was varied as 1 km, 3 km, 6 km, and 10 km, and the sources were placed such that no more than two sources existed within the same cell. Furthermore, it was assumed that all sources used the same frequency. A HAPS was placed at the center of the area, and localization simulations were performed for a total of 10 sources. The evaluation results are shown in Table 10 and Figure 21.

**Figure 22:**
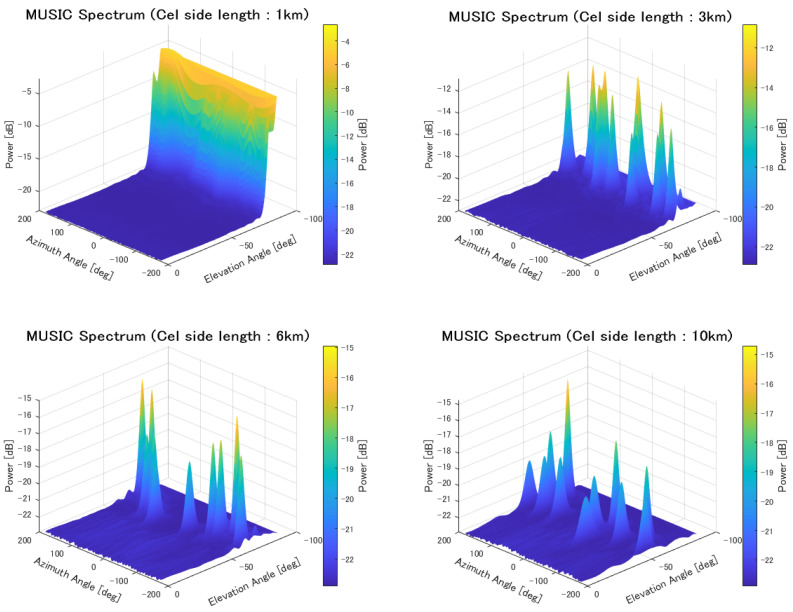
MUSIC spectrum in cell-based deployment with varying element spacing.

The MUSIC spectrum for each cell size is shown in Figure 22.

**Figure 23:**
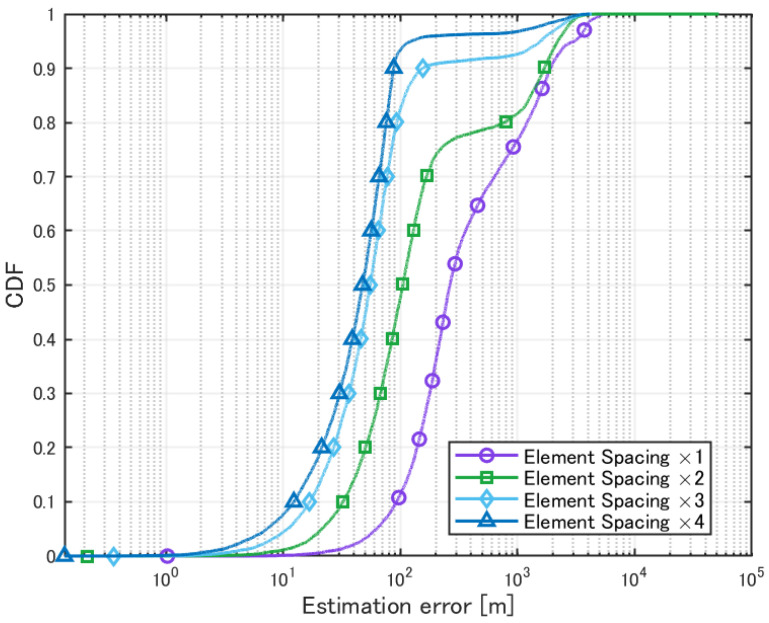
Cumulative distribution of estimation errors in cell-based deployment with varying element spacing.

Next, localization was performed under the condition that the side length of each cell was set to 1 km, while varying the element spacing from 1 to 4 times. The evaluation results are shown in Table 11 and Figure 23.

**Figure 24:**
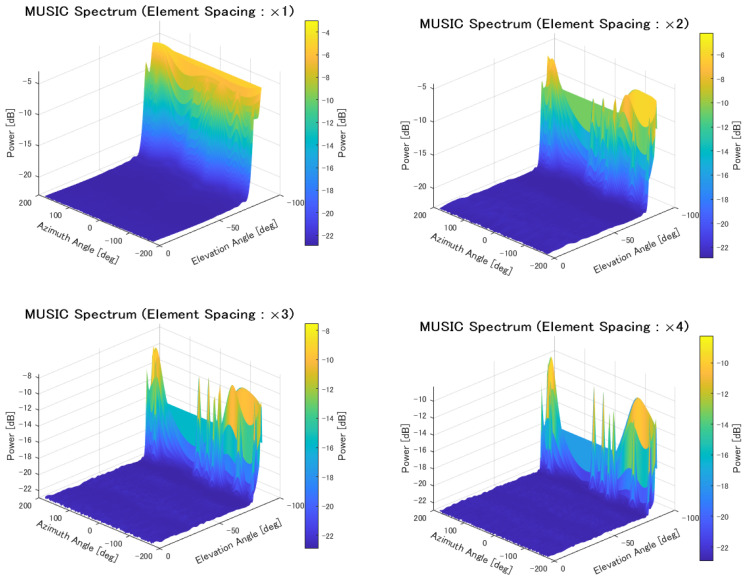
MUSIC spectrum in cell-based deployment with varying element spacing.

The MUSIC spectrum obtained for each element spacing is shown in Figure 24.

## 4. Discussion

### 4.1. Localization of a Single Source

In this study, we investigated the changes in estimation accuracy due to distance, the changes in estimation accuracy due to the transmission power of the source, the effectiveness of the proposed method against these factors, and the impact of considering edge diffraction around buildings.

When the horizontal distance from the HAPS to the source was within 60 km, the estimation error was within 1 km for almost all sources. On the other hand, in cases where the distance exceeded 80 km, significant degradation in accuracy was observed. This is likely because the direct wave could not reach the receiver, and the radio waves that arrived after multiple reflections were buried in noise power. Furthermore, when the distance was 100 km, the estimation error was within 1 km in less than 10% of the cases. This is because the elevation angle becomes nearly horizontal, causing deviations in height estimation to significantly affect horizontal distance estimation. Additionally, compared to localization over shorter distances, angle estimation errors have a greater impact on horizontal distance errors over longer distances.

When the transmission power of the source was reduced, a slight decrease in estimation accuracy was observed. On the other hand, adjusting the element panel’s tilt to modify the directivity improved the estimation accuracy. This is likely because the SNR of the signal increased. In the original element arrangement, radio waves from sources located downward were not sufficiently received, indicating that modifying the element arrangement is important.

When considering edge diffraction, no significant difference in estimation accuracy was observed at 5 km. However, at 20 km, a degradation in accuracy was confirmed. This is likely because considering edge diffraction increases the number of arriving radio waves. However, when the elevation angle is large, the variation in the angle of arrival is not significant, so the impact on accuracy is considered minimal. Additionally, even when direct or reflected waves do not reach the receiver, considering edge diffraction increases the likelihood of receiving an arriving wave, thereby improving the detection percentage.

### 4.2. Localization of Multiple Sources

Localization simulations were performed to investigate the differences in estimation accuracy depending on the number of sources, changes in estimation accuracy when the element spacing was varied, and mobile terminal estimation assuming a cell deployment by base stations.

As the number of sources increased, the accuracy worsened. In the MUSIC method, the direction of arrival is estimated using eigenvectors derived from the signal correlation matrix. Since an antenna array with 196 elements was used in this simulation, the number of eigenvectors available for direction estimation was reduced by the number of sources. As the number of sources increases, the number of available eigenvectors decreases, leading to a reduction in estimation accuracy. However, although the evaluation results appear to show a significant difference based on the number of sources, the cumulative distribution results do not reveal a substantial difference. This is likely due to the angle resolution. As shown in Figure 14, as the number of sources increases, the sharpness of the peaks decreases. As a result, when sources are closely spaced, the peaks overlap, and only a single peak may appear where two sources actually exist. Consequently, a small peak elsewhere may be incorrectly selected, leading to large errors, particularly when there are many sources.

To address this, we attempted to improve the angle resolution by increasing the element spacing. The evaluation results confirmed an improvement in the estimation accuracy. The cumulative distribution results also showed a reduction in poor accuracy cases. Increasing the element spacing increases the aperture length of the antenna, improving the angle resolution. As shown in Figure 15, increasing the element spacing improves the angle resolution. It was also confirmed that increasing the element spacing enables high estimation accuracy even when the number of sources is large.

However, increasing the element spacing causes grating lobes (aliasing of peaks), as observed in Figure 15. Although no significant degradation in accuracy was observed in this simulation, it is possible that differences in source power could have an impact. Furthermore, changing the element spacing is not as easy as tilting the element plate.

Therefore, we performed similar simulations by incorporating the directivity of each element into the steering vector. The estimation accuracy was improved similarly to when the element spacing was increased. Although the mean error worsened, this is likely because, in cases where estimation failed, the error was larger than in other scenarios. Additionally, two differences from the spectrum without directivity consideration were observed. First, the peak values corresponding to each source were nearly equal. Since the MUSIC method generates the spectrum using eigenvectors from the noise subspace, peak values are not affected by the source power. This suggests that incorporating directivity enables the pure noise components to be effectively used for direction estimation. Second, the peaks were sharper. In the spectrum without directivity consideration, the peaks were rounded. This is also likely due to the pure utilization of noise components. These results suggest that directivity should be considered. However, incorporating directivity requires real-time calculation of steering vectors that reflect the frequently changing element directivity. Although differences were observed in the spectrum, the evaluation results did not show a significant difference compared to varying element spacing. Therefore, it is necessary to verify the superiority of each method through experiments.

In the location estimation scenario assuming mobile terminal localization, where a total of 10 transmitters with the same frequency and power were deployed, one per cell, better results were obtained for larger cell sizes, as shown in Table 10. The reason for this is that, as previously mentioned, the smaller the cell size, the more likely the transmitters are to be located close to each other. Even in the absence of buildings, the estimation probability does not reach 100%, which is also attributed to the close proximity of the transmitters. Furthermore, the estimation accuracy for a cell size of 6 km per side was better than that for 10 km. This is because close-proximity situations occur less frequently, and the positioning error due to distance becomes a dominant factor.

From the cumulative distribution, when the cell size is small (particularly when the cell side length is 1 km), peak overlap occurs, making it difficult to accurately detect individual peaks.

When the element spacing was increased for localization in the case where the cell size was 1 km per side, the estimation results improved. As shown in Figure 24, it was confirmed that the resolution improvement allowed for better peak identification. The estimation probability also improved due to the influence of angular resolution. However, several cases were observed where the positioning error increased significantly. Specifically, when transmitters were in extremely close proximity, even if the number of transmitters was correctly estimated using the AIC method, the accuracy of angular resolution might not be sufficient to distinguish the peaks. When this phenomenon occurs, positioning accuracy deteriorates significantly, leading to cases where the positioning error becomes large.

## 5. Conclusions

This study evaluates the feasibility of radio source localization using a high-altitude-platform station (HAPS) at an altitude of 20 km, addressing the limitations of conventional ground-based receiving antennas in urban environments. Through numerical analysis, the effectiveness of HAPS-based localization is assessed for both single-source and multi-source scenarios under varying environmental conditions.

For single-source localization, results indicate that accuracy deteriorates with increasing horizontal distance. However, radio sources within 60 km could still be accurately localized, even in urban areas. Additionally, tilting antenna elements downward improved accuracy for low-power sources or those located below the HAPS. The study also found that edge diffraction from buildings had minimal impact on accuracy, while the overall detection percentage improved. These findings confirm that HAPS-based localization of a single source is feasible and reliable.

For multi-source localization, accuracy declined as the number of sources increased, particularly when sources were closely spaced, leading to reduced angle resolution. However, increasing element spacing and incorporating antenna directivity into the MUSIC method significantly enhanced localization accuracy. In scenarios involving mobile terminals with closely spaced, low-power sources, the system achieved sufficient estimation accuracy when cell side lengths were between 3 km and 10 km. However, at 1 km cell spacing, localization errors increased, though these were mitigated by further increasing the element spacing.

Overall, this study demonstrates the effectiveness of HAPS-based localization but highlights the need for higher angle resolution when sources are closely spaced, compared to ground-based systems. Additionally, antenna calibration and HAPS attitude adjustments were idealized in this study, and optimization of computation time and power consumption was not addressed. An initial study on these issues was conducted in [23], but further investigation will be carried out in our future work. Future research will also focus on real-world performance evaluations, practical calibration techniques, and computational efficiency enhancements to advance HAPS-based localization toward real-world deployment.

Furthermore, since our HAPS-based localization system achieves an estimation accuracy within several hundred meters, the possible location of the radio emitter can be significantly narrowed down. This suggests that conventional techniques, such as DEURAS or UAV-based systems, can then be further utilized to precisely pinpoint the source. Integrating these methods into a hybrid system will be explored in our future work.

## Figures and Tables

**Table 1:** Antenna specifications.

Parameter		Value
Tx	Center Frequency [GHz]	2
	Frequency Bandwidth [MHz]	10
	Transmission Power	Scenario dependent
	Antenna Type	Isotropic
	Antenna Height [m]	1
Rx	Antenna Type	cylindrical antenna
	Antenna Height [m]	20,000
	Number of Reflections	2

**Table 2:** Cylindrical antenna specifications.

Direction/Type	Item	Value
Horizontal	Number of Elements	31
	Element spacing	0.6λ
Vertical	Number of Elements	6
	Element spacing	0.5λ
Bottom	Number of Elements	10
	Element spacing	0.3λ

**Table 3:** Localization of a single source.

	5 km	10 km	20 km	40 km	60 km	80 km	100 km
Mean Error [m]	51.8	64.7	195.4	536.8	1149.1	2104.2	2496.6
CDF 90% [m]	87.7	113.6	193.7	479.6	935.5	1704.8	2807.4
Detection Percentage [%]	90.19	84.21	76.70	65.66	56.41	49.43	44.14

**Table 4:** Localization of a low-power single source.

	Type A [10 W]	Type A [200 mW]	Type B [200 mW]
Mean Error [m]	101.6	145.7	112.2
CDF 90% [m]	192.8	277.5	211.1
Detection Percentage [%]	76.51	75.08	76.44

**Table 5:** Localization of a single source considering edge diffraction.

	5 km	20 km
Mean Error [m]	52.9	368.2
CDF 90% [m]	95.5	251.1
Detection Percentage [%]	99.5	97.3

**Table 6:** Localization of multiple sources.

	10 Tx	20 Tx	30 Tx	40 Tx
Mean Error [m]	1717.9	3645.9	8142.2	11579
CDF 50% [m]	337.7	493.7	767.2	1093.8
CDF 90% [m]	2017.5	3932.7	7384.9	1328.9
Detection Percentage [%]	99.85	99.62	99.50	99.09

**Table 7:** Localization of multiple sources with varying element spacing.

	×1	×2	×3	×4
Mean Error [m]	1717.9	516.4	411.8	360.8
CDF 50% [m]	337.7	277.6	256.5	248.1
CDF 90% [m]	2017.5	918.0	788.7	754.5
Detection Percentage [%]	99.85	99.94	99.97	99.98

**Table 8:** Localization with varying element spacing (40 transmission sources).

	×1	×4
Mean Error [m]	11579	659.8
CDF 50% [m]	1093.8	264.5
CDF 90% [m]	13289	886.8
Detection Percentage [%]	99.09	99.91

**Table 9:** Localization using the MUSIC method incorporating element directivity.

	Directivity Considered Element Spacing: ×1	Directivity Not Considered Element Spacing: ×2	Directivity Not Considered Element Spacing: ×1
Mean Error [m]	2073.4	516.4	1717.9
CDF 50% [m]	252.8	277.6	337.7
CDF 90% [m]	825.7	918.0	2017.5
Detection Percentage [%]	99.88	99.94	99.85

**Table 10:** Localization of sources in cell-based deployment.

	1 km	3 km	6 km	10 km
Mean Error [m]	710.7	415.6	155.5	123.9
CDF 50% [m]	264.9	73.8	60.0	72.8
CDF 90% [m]	1909.4	232.7	125.7	175.8
Detection Percentage [%]	92.74	99.98	100	100

**Table 11:** Localization of signal sources in cell-based deployment with varying element spacing.

	×1	×2	×3	×4
Mean Error [m]	710.7	456.9	207.5	116.8
CDF 50% [m]	264.9	105.1	55.1	47.5
CDF 90% [m]	1909.4	1692.9	154.7	87.7
Detection Percentage [%]	92.74	99.80	99.98	99.99

## Data Availability

Data are contained within the article.
